# Diminished Social Motivation Negatively Impacts Reputation Management: Autism Spectrum Disorders as a Case in Point

**DOI:** 10.1371/journal.pone.0031107

**Published:** 2012-01-27

**Authors:** Coralie Chevallier, Catherine Molesworth, Francesca Happé

**Affiliations:** 1 MRC Social, Genetic and Developmental Psychiatry Centre, Institute of Psychiatry, King's College London, London, United Kingdom; 2 Center for Autism Research, University of Pennsylvania and Children's Hospital of Philadelphia, Philadelphia, Pennsylvania, United States of America; French National Centre for Scientific Research, France

## Abstract

Human beings are endowed with a unique motivation to be included in social interactions. This natural social motivation, in turn, is thought to encourage behaviours such as flattery or self-deprecation aimed to ease interaction and to enhance the reputation of the individual who produces them. If this is the case, diminished social interest should affect reputation management. Here, we use Autism Spectrum Disorders (ASDs) –primarily characterised by pervasive social disinterest– as a model to investigate the effect of social motivation on reputation management. Children first rated a set of pictures and were then given the opportunity to inflate their initial ratings in front of an experimenter who declared that she had drawn the picture. Contrary to the controls, children with ASD did not enhance their ratings in the drawer's presence. Moreover, participants' flattery behaviour correlated with self-reports of social enjoyment. Our findings point to a link between diminished social interest and reputation management.

## Introduction

The intensity of cooperative activities is a defining feature of our species' ecological niche. Trading, hunting, gathering, and all other types of collective actions give access to benefits that would have otherwise remained beyond our limits. As a consequence of this evolutionary history, the drive to be included in the most fruitful cooperative ventures is central to human psychology [1]. Two intertwined psychological dispositions are of special importance in this respect: the motivation to engage in social interactions and the ability to present oneself as a likable partner in these interactions. Failure to achieve either of these goals might result in being isolated, thereby loosing the potential benefits of cooperation (for experimental evidence, see e.g. [2]). As a result, most people exhibit clear signs that they find social interactions rewarding and that they therefore care about their reputation.

Concern for reputation is mostly expressed through ingratiating behaviours, such as downward self-presentation (e.g., apology, modesty, self-deprecation) and other-enhancement (e.g., flattery). These behaviours aim to elicit positive attitudes in the recipient, thereby enhancing the reputation of the ingratiator. A substantial amount of theory and empirical data suggests that flattery is one of the most powerful forms of ingratiation and that it is an effective means of producing positive effects in the target (e.g., perceived likeability, perceived competency, hiring decisions, tipping, pay raises, and so on); for a meta-analytic review of 69 studies, see [3], see also [4]. It is important to note that, as suggested by Jones and Wortman [5], “ingratiating overtures are rarely the result of conscious or deliberate tactical planning”. Rather, ingratiation should be seen as a spontaneous bias, a natural propensity to try and enhance one's image in front of others, without necessarily consciously aiming to do so.

Ingratiation in children is less well documented but there is evidence that children as young as 2 to 3 years of age spontaneously engage in strategies of self-enhancement. For instance, they share their successes with others more frequently than they share their failures [6] and they present themselves in overly positive lights when describing their conflicts with siblings [7]. In preschool years, children also become able to engage in relatively subtle forms of pro-social lies, like, for instance, telling an experimenter that they look good for a picture when they have in fact a conspicuous mark of lipstick on the nose [8]. They can also mask a disappointed emotional expression in the presence of an experimenter who gives them an undesirable gift [9], or declare that they are happy with an undesirable gift for politeness purposes [10]. Later on in development (at about 7 to 9 years of age), children become able to acknowledge explicitly that politeness sometimes trumps honesty [11].

If social motivation underlies reputation management, then one would predict reduced or absent ingratiation, flattery and other types of ‘social grooming’ in individuals with diminished social interest. In autism spectrum disorders (ASD), social motivation is diminished or atypical. Lack of social orienting is one of the earliest symptoms of ASD [12], [13], [14], [15] and is believed by many to be the primary deficit in autism [12], [16], [17]. Numerous studies, using a variety of techniques, have demonstrated a lack of preferential attention for social stimuli: preference for non social contingencies over biological motion [13], for non-speech signals over motherese [18], and for objects over people [15], [19], [20]. Social engagement is also markedly decreased in ASD: The earliest signs of autism include a decreased response to one's own name [21], diminished orientation to social stimuli in general [15], rare sharing gestures such as pointing or showing [22], and, later on in development, fewer responses to others' bids for joint attention [23] and diminished interest in collaborative activities [24].

To test the links between social motivation and reputation management, we used a simple paradigm in which participants are given the opportunity to flatter another person: Recent findings by Fu and Lee [25] indicate that 4- to 6-year-olds spontaneously improve their rating of a drawing in the presence of the artist, demonstrating their command of other-enhancement strategies. Aside from its simplicity and ecological value, this task was chosen because there is evidence that it truly targets flattery behaviours. In a follow-up experiment using the same procedure, Fu and Lee indeed demonstrated that 6-year-olds enhance their ratings to a greater extent for individuals with whom they are likely to interact in the future than for those they are uncertain to encounter again. This pattern of results thus carries the signature of flattery, which as the authors argue, “is effective for maintaining and enhancing existing relationships” (p. 261). In this paper, we concentrated on whether adolescent with ASD improve their rating of a drawing in the presence or absence of the artist using Fu and Lee's original setting. We predicted that, unlike typically developing children, children with ASD would not be prone to this flattery bias, and would not alter their ratings to please the artist. We further hypothesized that flattery scores (positive change in picture ratings) would correlate with an independent measure of social enjoyment.

## Methods

### Ethics statement

The procedure was approved by the local ethics committee (PNM/09/10-8, Psychiatry, Nursing & Midwifery Research Ethics Sub-Committee,King's College London). Parents of all participants gave their written informed consent prior to our coming to the school and children gave informed assent prior to the beginning of the procedure.

### Participants

Thirty-six male adolescents (18 with ASD and 18 Typically Developing, henceforth TD) took part in the study. The ASD and the control groups were matched on chronological age and IQ, as assessed with the Wechsler Abbreviated Scales of Intelligence, two-subtest form [26] (see Table 1). Children in the ASD group were recruited from special education schools or unit. All had received a formal diagnosis of an ASD by an independent clinician, according to the standard Diagnostic and Statistical Manual of Mental Disorders-IV criteria (APA, 1994) and all were high functioning. Eleven participants had received a diagnosis of Asperger Syndrome (AS) and seven of autism. In addition to this diagnostic information, we used the Autism Diagnostic Observational Schedule [27] to further characterize the current profile of the participants (see Table 1): 11 participants scored above ADOS cut-off for autism, 4 scored above ADOS cut-off for ASD, 3 scored above cut-off in only one of the two subscales, no participant was below cut-off in both subscales (see Appendix S1). Omitting the 3 participants whose total ADOS score fell below cut-off for ASD did not alter the results, and data are reported below for all 18 participants in the ASD group. The TD controls were recruited in mainstream schools and had no identified special needs.

**Table 1:** Participants' mean age, IQ and ADOS-G scores in the ASD group.

	ASD	TD	t(df) value, *p* value
mean age ± sd	13;8±0;10	13;11±0;10	t(34) = −0.95, *p* = .35
Age range	12;4–15;1	12;10–15;9	
mean IQ ± sd	102±15	103±14	t(34) = 0.30, *p* = .77
mean ADOS ± sd	11.2±4.3	NA	

### Stimuli

**Figure 1:**
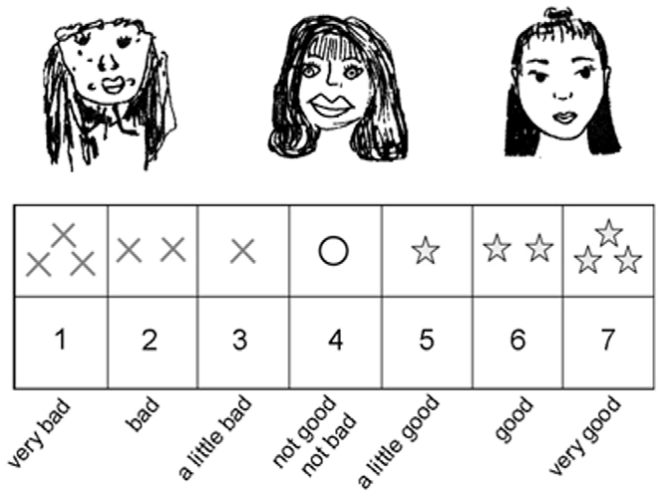
Seven-point Likert scale used to guide children's ratings and one example of a drawing of poor, medium and high quality (from left to right).

The drawings were chosen from a collection of children's self-portraits (line drawings in black on a white background) to include drawings of poor, medium and high quality. We then asked 10 adult participants to rate this collection of 29 drawings on a seven-point Likert scale ranging from 1 (very bad) to 7 (very good) and selected the thirteen drawings that elicited the strongest levels of agreement. Four drawings were included in the ‘poor quality’ category, five in the ‘medium quality’ category, and four in the ‘high quality’ category. The scale used for children combined verbal, graphic and numerical cues (see Figure 1).

### The pleasure scale for children

The pleasure scale is a validated instrument used to assess anhedonia in children [28]. It consists of 39 items pertaining to physical, social, or other sources of pleasure (see (1–3) for an example in each category and [28] for a complete list).

1. You are cycling down the street very fast while still in good control of yourself.
2. You accidentally overhear your teacher telling the principal what a terrific student you are.
3. On a Saturday night, you stay up watching television as long as you want.

The child is read each item out loud and asked to rate the situation on a 3-point Likert scale: 1 for ‘Very happy’, 2 for ‘Happy’ and 3 for ‘Neither happy nor unhappy’. Thus, high scores reflect diminished pleasurable responses (or increased anhedonia).

### Procedure

Children were tested individually in a quiet room. Two experimenters (E1 and E2) were involved in the procedure. Whilst E2 was waiting outside the testing room, E1 introduced the child to the scale and presented three practice drawings. In the test phase, E1 asked the child to rate the remaining ten drawings one by one. E1 then placed two of the drawings for which the child had provided a medium rating at the bottom of the stack and left the room declaring that the experiment was over and that E2 would now come to do a few more things with the child. The two drawings placed at the bottom of the stack were the ones which the child would have to rate again and were chosen in the middle range in order to allow children to flexibly increase or decrease their scores.

E2 came in the room and asked the child what he had been up to with E1. Upon his response, E1 took the pile of drawings and declared: ‘Oh, so you were ratings these drawings, that's interesting!’, whilst casually looking through the pile. When E1 got to the penultimate and last drawing, she asked the child for a second rating. In the control condition, she would simply say: ‘So how much do you think this one should get?’. In the experimental condition, she would say: ‘Oh, that's my drawing! How much do you think this one should get?’ The order of the control and experimental rating was counterbalanced across participants. E2 then went on presenting an unrelated task (WASI), filled out the pleasure scale and debriefed the children.

### Data analysis

The data were analysed using Statistica 7.0. Shapiro Wilk tests revealed that the data were not normally distributed. Non parametric statistics were therefore used throughout the analyses. All *p* values assume two-tailed tests.

## Results

We first checked whether the two groups differed in their overall appreciation of the drawings by comparing their average first ratings to all ten drawings. We found that the ASD group and the TD group gave comparable ratings, *U* = 115.0, *Z* = 1.49, *p* = .14, Mann-Whitney U Test, which suggests that neither group was harsher or more generous overall than the other (see Table 2).

**Table 2:** Descriptive statistics in the ASD and TD groups for all variables of interest (First rating, Difference score for the control drawing and Difference score for the experimental drawing).

		median (min – max)	mean ± sd	95% confidence
ASD	First Ratings	4.15 (3.00–5.10)	4.06±0.64	3.74–4.38
	Diff. Score Control	0.00 (−1.00–1.00)	−0.17±0.62	−0.47–0.14
	Diff. Score Experimental	0.00 (−3.00–3.00)	0.11±1.71	−0.74–0.96
TD	First Ratings	3.90 (−2.90–4.50)	3.77±0.53	3.50–4.03
	Diff. Score Control	0.00 (−2.00–2.00)	0.03±1.09	−0.51–0.57
	Diff. Score Experimental	1.00 (0.00–2.00)	1.00±0.66	0.67–1.33

**Figure 2:**
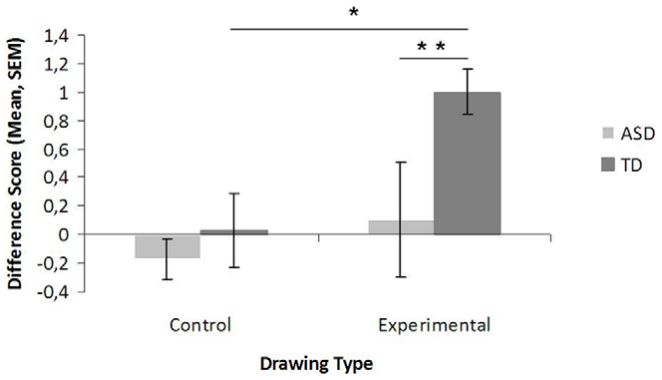
Difference scores in the Control and Experimental conditions for the ASD (light grey) and the TD group (dark grey). Mean and SEM are depicted. * indicates p values≤.05, ** indicates p values≤.01.

The crucial dependent variable was the difference score between the first and the second rating for both Drawing types (Control vs. Experimental). In the Control condition, both groups kept their rating constant, and the difference score did not differ from zero, Z_ASD_ = 1.01, *p* = .31; Z_TD_ = 0.06, *p* = .95, one-sample Wilcoxon signed rank test. In the Experimental condition, however, the difference score did not differ from zero in the ASD group, Z = 0.38, *p* = .71, but did in the TD group, Z = 3.47, *p* = .0005, one-sample Wilcoxon signed rank test (see Figure 2 and Table 2).

Between-group comparisons further revealed that both groups were comparable in the Control condition, *U* = 138.5, *Z* = −.82, *p* = .41; but that the TD group had significantly larger difference scores in the Experimental condition than the ASD group, *U* = 102.0, *Z* = −1.94, *p* = .05, Mann-Whitney U Test (see Figure 2 and Table 2).

Finally, the comparison between the difference score for the Experimental drawing was significantly larger than for the control drawing in the TD group, but not in the ASD group: TD group, Z = 2.73, *p* = .006, ASD Z = 0.51, p = .61, Wilcoxon matched pairs test (see Figure 2 and Table 2).

In line with our predictions, across all participants the social sub-score of the pleasure scale was significantly negatively correlated with the difference score in the experimental condition: the higher the score in social anhedonia, the lower the score in flattery (see Table 3). In other words, social enjoyment was associated with increased flattery behaviour. This was confirmed when both groups were considered separately, with a significant correlation in the ASD group, and a similar trend in the TD group (see Table 3). By contrast, the other two sub-scores were not associated with flattery.

**Table 3:** Correlations between the difference score in the experimental condition and social, physical and other sources of pleasure.

	Social pleasure		Physical pleasure		Other sources of pleasure	
	mean ± sd	r	mean ± sd	r	mean ± sd	r
*All participants*	*34.7±7.2*	*−0.53* **	*14.5±2.6*	*−0.09*	*20.7±3.8*	*−0.27*
ASD	36.6±8.3	−0.52*	14.2±2.4	−0.18	20.2±4.1	−0.43∼
TD	32.9±5.6	−0.42∼	14.7±2.8	0.07	21.2±3.6	0.01

*Indicates p values≤.05,

**indicates p values≤.01,

∼ indicates p values≤.10.

## Discussion

In this paper, we used ASD as a model to explore the relationship between social motivation and flattery. Participants first rated a set of pictures and were then given the opportunity to inflate their initial ratings in front of an experimenter who declared that she had drawn the picture. In line with our predictions, we demonstrated that children with an ASD did not enhance their ratings in the presence of the drawer: There was no significant difference between their initial rating and their second rating, and the resulting difference score was similar to that obtained in the control condition (judging a picture in the artist's absence). In the TD group, by contrast, children increased their ratings by about one point in the experimental condition: the difference score in this condition differed significantly from zero, from the control condition, and from the Difference Score obtained in the ASD group. Using an anhedonia scale, we further demonstrated that participants' flattery behaviour correlated with their self-report of enjoyment of social interactions.

Though this is the first direct investigation of the link between diminished social interest and reputation management, a couple of recent studies also suggest that autism provides a good model to understand the processes by which people establish, maintain, and enhance their relationships with others. In particular, in a study addressing the understanding and use of self-presentational display rules, children with ASD were found to be less able to refrain from expressing their emotions in order to deceive an experimenter [29]. Similarly, in a study examining the acoustics of laughter during play interactions, Hudenko et al. demonstrated that children with autism express laughter primarily in response to positive internal states rather than for more social purposes [30]. More generally, children with autism are known to share fewer smiles during social interactions than their TD peers; e.g., [31], [32].

These results also fit with clinical and parental accounts that emphasise the –often blunt– honesty with which individuals with autism express their opinions. Kanner's initial description of the disorder [33], for instance, included a number of statements about his patients' disregard for others' opinion (e.g., his notes about case 5: “No competitive spirit, no desire to please her teacher. If she knew more than any member in the class about something, she would give no hint of it, just keep quiet, maybe not even listen”). In a self-help book written by a mother of a child with Asperger syndrome (AS), reputation management is also mentioned as an area of special challenge and parents are encouraged to appreciate their child's honesty as a special attribute: “Often the ‘rude’ behaviour of a child with AS is really just honest behaviour. Honesty and directness are very much a part of Asperger (…). Looked at it positively, it's very rare and refreshing to come across the lack of hypocrisy and pretence which is so typical with AS” [34].

The question of what accounts for diminished concern for reputation in ASD is, however, unresolved. Individuals with autism may indeed fail to flatter others because they lack the ability to empathize with others' emotions or to appreciate that a negative rating might elicit negative emotions in the recipient. Such a mentalistic interpretation was recently put forward in a study revealing that adults with the condition are not influenced by the presence of an observer when asked to make a charitable donation [35]. According to Izuma et al., this absence of audience effects in autism points to a specific deficit in taking into account one's reputation *in the eyes of others*. In a recent review on the topic, Tennie, Frith and Frith [36] also suggested that mastery of reputation management strategies derives from ToM.

But does mentalizing suffice to sustain efficient reputation management? Conceptually, it appears that both mechanisms are relatively distinct: One might indeed have a good understanding of others' mental states yet no interest in putting this knowledge to use to optimize social relationships. Whether or not people use the output of mentalizing cognitive modules to enhance their image is indeed likely to depend on individual psychological differences (i.e., one's general concern for others' opinion and social approval) and the specific circumstances of the interaction (e.g., how much one cares about a particular person's opinion). As a consequence, understanding what others want and expect might end up being of little use without the basic drive to act in accordance with these desires and expectations. If it appears that mentalizing is not *sufficient* to account for reputation management, the next question is whether it is at all *necessary*. Animal research suggests that the answer to this question is not straightforward. Several examples of audience effects and tactical deception have indeed been reported among reef fish, a species which is arguably not equipped with higher cognitive modules such as ToM; for a short review, see [36]. However, whether reputation in human and non human animals relies on the same mechanisms remains unknown. One could indeed imagine that humans, being especially apt at mentalizing, spontaneously put these skills to the service of reputation management.

In autism then, it remains unclear whether deficits in reputation management result from 1) impaired ToM, 2) diminished social motivation, or 3) a combination of both. In the present study, the correlation between social anhedonia and flattery behaviour can be seen as a first step in exploring the social motivation hypothesis and echoes classic findings showing that individuals scoring high in need for social approval donate more money to charity [37]. However, due to its correlational nature, this finding is to be interpreted with caution, and the methods used in the current study do not provide a definite way of supporting one interpretation over the other. Another limitation of our study is that our sample consisted of boys only, which precludes investigating possible gender differences in the development of reputation management. This caveat is particularly important to highlight given that females have been shown to score higher in some social questionnaires, such as the empathy quotient [38].

Despite these limitations, these findings raise the exciting question of how much inter-individual differences in social interest play a role in social skills. In the present study, the correlation between social anhedonia and flattery behaviour can be seen as a first step in exploring this issue. However, a more systematic investigation of the modulating influence of social interest on behaviours aimed at making oneself noticed, valued, and accepted remains to be carried out. Indeed, although there are good evolutionary reasons to posit that the drive to have good social relationships is universal, this ‘need to belong’ [39] is likely to vary in the general population (just like height, intelligence, or verbal fluency). Autism can thus be seen as the extreme end of a continuum ranging from low to high need to belong –an extreme case of diminished social interest [17], [40]– and can therefore function as an insightful model to understand humans' deep-seated drive to seek acceptance and avoid rejection.

## Supporting Information

Appendix S1 Individual diagnostic information in the ASD group. Diagnosis refers to the clinical assessment provided by a psychologist or psychiatrist as recorded on school files. Scores on the ADOS-G are derived from the diagnostic algorithm and represent the current profile of the participant. Cut-off points for autism and ASD are set at 10 and 7 respectively for the total score, 3 and 2 for the communication subscale, and 6 and 4 for the social interaction subscale. This table also presents individual difference scores in the experimental condition.
(DOC)

## References

[1] Tomasello M, Carpenter M, Call J, Behne T, Moll H (2005). Understanding and sharing intentions: The origins of cultural cognition.. Behavioral and Brain Science.

[2] Pradel J, Euler H, Fetchenhauer D (2009). Spotting altruistic dictator game players and mingling with them: the elective assortation of classmates.. Evolution and Human Behavior.

[3] Gordon R (1996). Impact of Ingratiation on Judgements and Evaluations: A Meta-Analytic Investigation* 1.. Journal of Personality and Social Psychology.

[4] Higgins C, Judge T, Ferris G (2003). Influence tactics and work outcomes: a meta analysis.. Journal of Organizational Behavior.

[5] Jones E, Wortman C (1973). Ingratiation: An attributional approach..

[6] Stipek D, Recchia S, McClintic S, Lewis M (1992). Self-Evaluation in Young Children.. Monographs of the Society for Research in Child Development.

[7] Ross H, Smith J, Spielmacher C, Recchia H (2004). Shading the Truth: Self-Serving Biases in Children's Reports of Sibling Conflicts.. Merrill-Palmer Quarterly.

[8] Talwar V, Lee K (2002). Emergence of white-lie telling in children between 3 and 7 years of age.. Merrill Palmer Quarterly.

[9] Cole PM (1986). Children's Spontaneous Control of Facial Expression.. Child Development.

[10] Talwar V, Murphy S, Lee K (2007). White lie-telling in children for politeness purposes.. International journal of behavioral development.

[11] Xu F, Bao X, Fu G, Talwar V, Lee K (2010). Lying and Truth Telling in Children: From Concept to Action.. Child Development.

[12] Dawson G, Meltzoff A, Osterling J, Rinaldi J, Brown E (1998). Children with Autism Fail to Orient to Naturally Occurring Social Stimuli.. Journal of Autism and Developmental Disorders.

[13] Klin A, Lin D, Gorrindo P, Ramsay G, Jones W (2009). Two-year-olds with autism orient to non-social contingencies rather than biological motion.. Nature.

[14] Osterling J, Dawson G, Munson J (2002). Early recognition of 1-year-old infants with autism spectrum disorder versus mental retardation.. Development and Psychopathology.

[15] Maestro S, Muratori F, Cavallaro M, Pecini C, Cesari A (2005). How Young Children Treat Objects and People: An Empirical Study of the First Year of Life in Autism.. Child Psychiatry and Human Development.

[16] Klin A, Jones W, Schultz R, Volkmar F (2003). The enactive mind, or from actions to cognition: lessons from autism.. Philosophical Transactions: Biological Sciences.

[17] Schultz R (2005). Developmental deficits in social perception in autism: the role of the amygdala and fusiform face area.. International Journal of Developmental Neuroscience.

[18] Kuhl P, Coffey-Corina S, Padden D, Dawson G (2005). Links between social and linguistic processing of speech in preschool children with autism: behavioral and electrophysiological measures.. Developmental Science.

[19] Klin A, Jones W, Schultz R, Volkmar F, Cohen D (2002). Visual Fixation Patterns During Viewing of Naturalistic Social Situations as Predictors of Social Competence in Individuals With Autism.. Archives of General Psychiatry.

[20] Swettenham J, Baron-Cohen S, Charman T, Cox A, Baird G (1998). The frequency and distribution of spontaneous attention shifts between social and nonsocial stimuli in autistic, typically developing, and nonautistic developmentally delayed infants.. Journal of Child Psychology and Psychiatry.

[21] Osterling J, Dawson G (1994). Early recognition of children with autism: A study of first birthday home videotapes.. Journal of Autism and Developmental Disorders.

[22] Wetherby AM, Woods J, Allen L, Cleary J, Dickinson H (2004). Early Indicators of Autism Spectrum Disorders in the Second Year of Life.. Journal of Autism and Developmental Disorders.

[23] Leekam S, Baron-Cohen S, Perrett D, Milders M, Brown S (1997). Eye-direction detection: A dissociation between geometric and joint attention skills in autism.. British Journal of Developmental Psychology.

[24] Liebal K, Colombi C, Rogers S, Warneken F, Tomasello M (2008). Helping and cooperation in children with autism.. Journal of Autism and Developmental Disorders.

[25] Fu G, Lee K (2007). Social grooming in the kindergarten: the emergence of flattery behavior.. Developmental Science.

[26] Wechsler D (1999). Wechsler Abbreviated Scales of Intelligence.

[27] Lord C, Risi S, Lambrecht L, Cook EH, Leventhal BL (2000). The Autism Diagnostic Observation Schedule—Generic: A Standard Measure of Social and Communication Deficits Associated with the Spectrum of Autism.. Journal of Autism and Developmental Disorders.

[28] Kazdin A (1989). Evaluation of the Pleasure Scale in the assessment of anhedonia in children.. Journal of the American Academy of Child & Adolescent Psychiatry.

[29] Barbaro J, Dissanayake C (2007). A Comparative Study of the Use and Understanding of Self-Presentational Display Rules in Children with High Functioning Autism and Asperger's Disorder.. Journal of Autism and Developmental Disorders.

[30] Hudenko W, Stone W, Bachorowski J (2009). Laughter Differs in Children with Autism: An Acoustic Analysis of Laughs Produced by Children With and Without the Disorder.. Journal of Autism and Developmental Disorders.

[31] Kasari C, Sigman M, Mundy P, Yirmiya N (1990). Affective sharing in the context of joint attention interactions of normal, autistic, and mentally retarded children.. Journal of Autism and Developmental Disorders.

[32] Reddy V, Williams E, Vaughan A (2002). Sharing humour and laughter in autism and Down” s syndrome.. British Journal of Psychology.

[33] Kanner L (1943). Autistic Disturbances of Affective Contact.. Nervous Child.

[34] Boyd B (2003). Parenting a Child With Asperger Syndrome: 200 Tips and Strategies.

[35] Izuma K, Matsumoto K, Camerer C, Adolphs R (2011). Insensitivity to social reputation in autism.. Proceedings of the National Academy of Sciences of the United States of America.

[36] Tennie C, Frith U, Frith C (2010). Reputation management in the age of the world-wide web.. Trends in Cognitive Sciences.

[37] Satow K (1975). Social Approval and Helping.. Journal of Experimental Social Psychology.

[38] Baron-Cohen S, Wheelwright S (2004). The Empathy Quotient: An Investigation of Adults with Asperger Syndrome or High Functioning Autism, and Normal Sex Differences.. Journal of Autism and Developmental Disorders.

[39] Baumeister RF, Leary MR (1995). The need to belong: desire for interpersonal attachments as a fundamental human motivation.. Psychological Bulletin.

[40] Dawson G, Munson J, Estes A, Osterling J, McPartland J (2002). Neurocognitive function and joint attention ability in young children with autism spectrum disorder versus developmental delay.. Child Development.

