# Supplementary material for: Diminished Social Motivation Negatively Impacts Reputation Management: Autism Spectrum Disorders as a Case in Point
Source: PLoS One. 2012 Jan 27;7(1):e31107. doi: 10.1371/journal.pone.0031107 (PMC3267764; doi:10.1371/journal.pone.0031107)
Supplement: Appendix S1 — Individual diagnostic information in the ASD group. Diagnosis refers to the clinical assessment provided by a psychologist or psychiatrist as recorded on school files. Scores on the ADOS-G are derived from the diagnostic algorithm and represent the current profile of the participant. Cut-off points for autism and ASD are set at 10 and 7 respectively for the total score, 3 and 2 for the communication subscale, and 6 and 4 for the social interaction subscale. This table also presents individual difference scores in the experimental condition. (DOC) [file pone.0031107.s001.doc]

| **Participant** | **Diagnosis** | **ADOS total** | **Communication** | **Social interaction** | **Difference score** |
| --- | --- | --- | --- | --- | --- |
| 1 | ASD | 13 | 4 | 9 | 0 |
| 2 | AS | 12 | 2 | 10 | -1 |
| 3 | AS | 14 | 4 | 10 | -1 |
| 4 | AS | 16 | 3 | 13 | 0 |
| 5 | ASD | 17 | 4 | 13 | 2 |
| 6 | ASD | 7 | 3 | 4 | 3 |
| 7 | ASD | 14 | 3 | 11 | -1 |
| 8 | ASD | 6 | 1 | 5 | 1 |
| 9 | AS | 17 | 3 | 14 | -1 |
| 10 | AS | 10 | 4 | 6 | -3 |
| 11 | ASD | 18 | 4 | 14 | 0 |
| 12 | ASD | 8 | 2 | 6 | -2 |
| 13 | AS | 5 | 1 | 4 | 0 |
| 14 | AS | 13 | 4 | 9 | 3 |
| 15 | AS | 8 | 0 | 8 | -1 |
| 16 | AS | 7 | 2 | 5 | 2 |
| 17 | AS | 6 | 3 | 3 | 2 |
| 18 | AS | 11 | 3 | 8 | -1 |
